# Supplementary material for: LRR Receptor-like Protein in Rapeseed Confers Resistance to Sclerotinia sclerotiorum Infection via a Conserved SsNEP2 Peptide
Source: Int J Mol Sci. 2025 May 10;26(10):4569. doi: 10.3390/ijms26104569 (PMC12110989; doi:10.3390/ijms26104569)
Supplement: Supplementary file 1 [file ijms-26-04569-s001.zip › ijms-3603879-supplementary.pdf]

Supplementary Information

1 Supplementary Figures

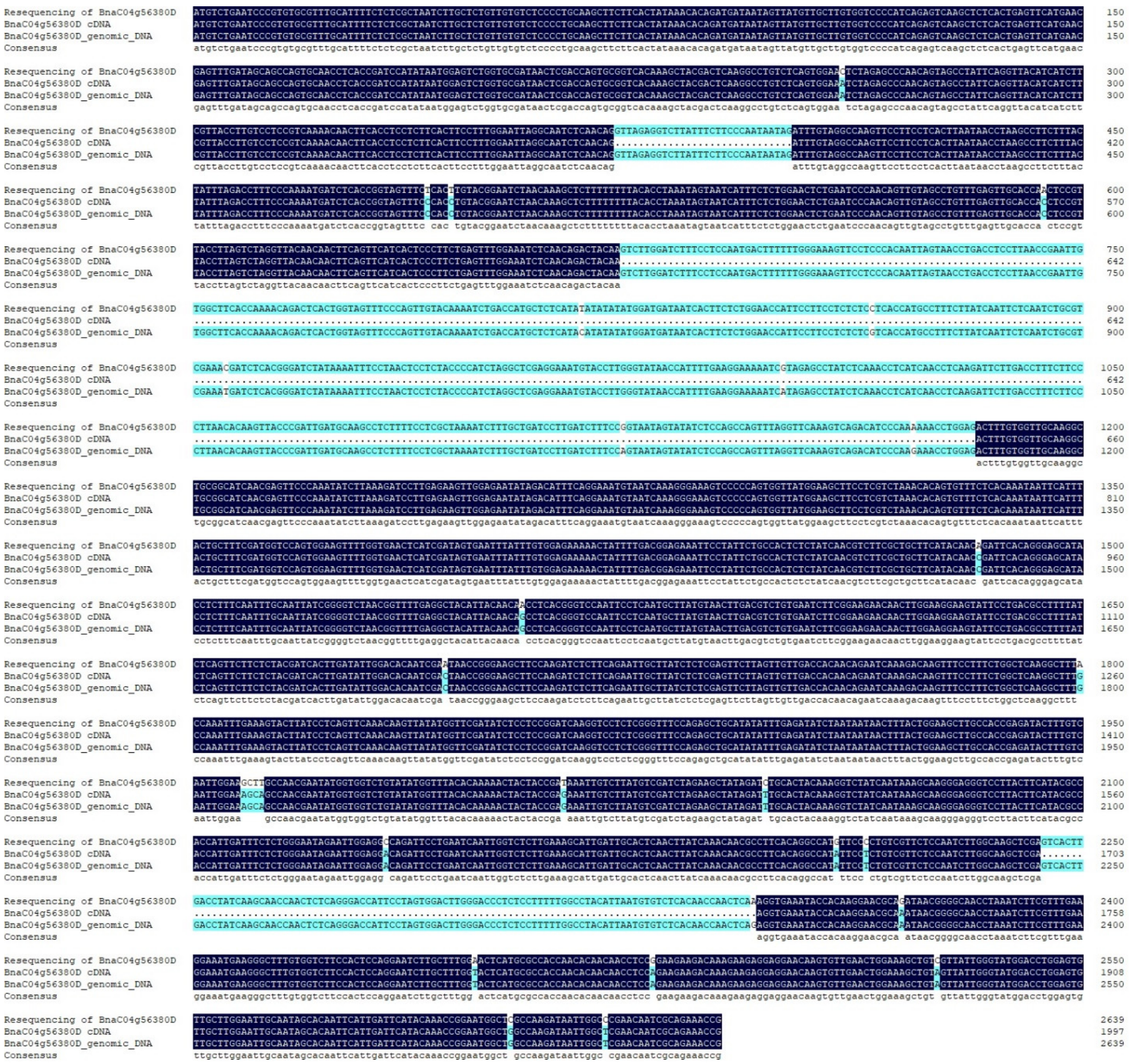

Supplementary Figure S1. Alignments of the resequencing sequence of *BnaC04g56380D*. The first row was re-sequencing results of *BnaC04g56380D* (the amplified sample was derived from RNA of XY15), the second row was cDNA sequences of *BnaC04g56380D* in the database, and the third row was genomic DNA sequences of *BnaC04g56380D* in the database.

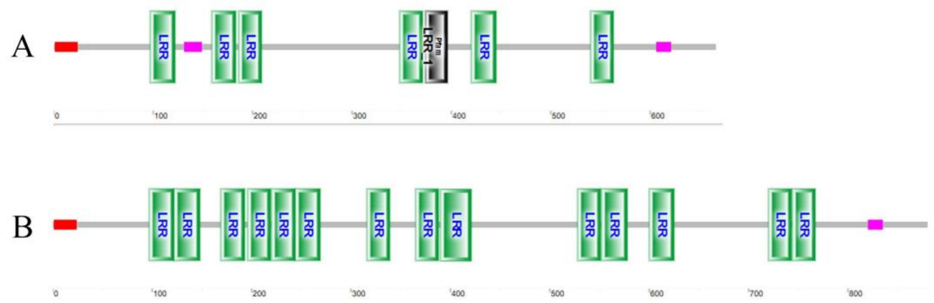

Supplementary Figure S2. Secondary structure of *Bna*C04g56380D. (A) Prediction results for the *Bna*C04g56380D sequence obtained from the Ensembl plants. (B) Prediction results for the *Bna*C04g56380D sequence cloned from XY15 and resequenced. The red box indicates the signal peptide, the green boxes represent the leucine-rich repeats (LRR) domain, and the purple box indicates the low complexity region.

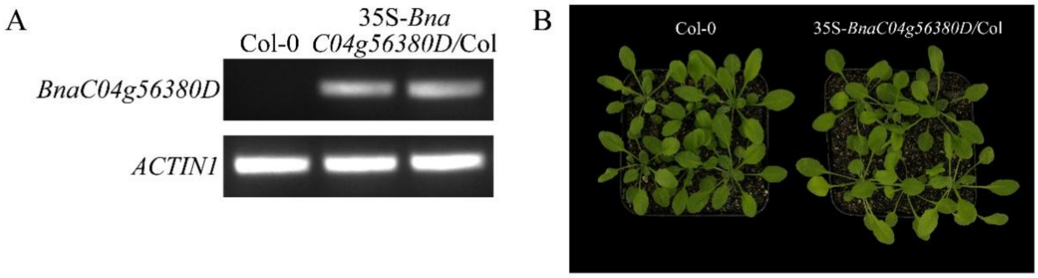

Supplementary Figure S3. *Bna*C04g56380D overexpression in Col-0. (A) Genotype identification of overexpression lines was performed using semi-quantitative PCR of *Bna*C04g56380D, *ACTIN1* as the reference gene. (B) Phenotype of 35S- *Bna*C04g56380D /Col and Col-0, all plants were grown on soil at 22°C in parallel and photographed when they were 4 weeks old.

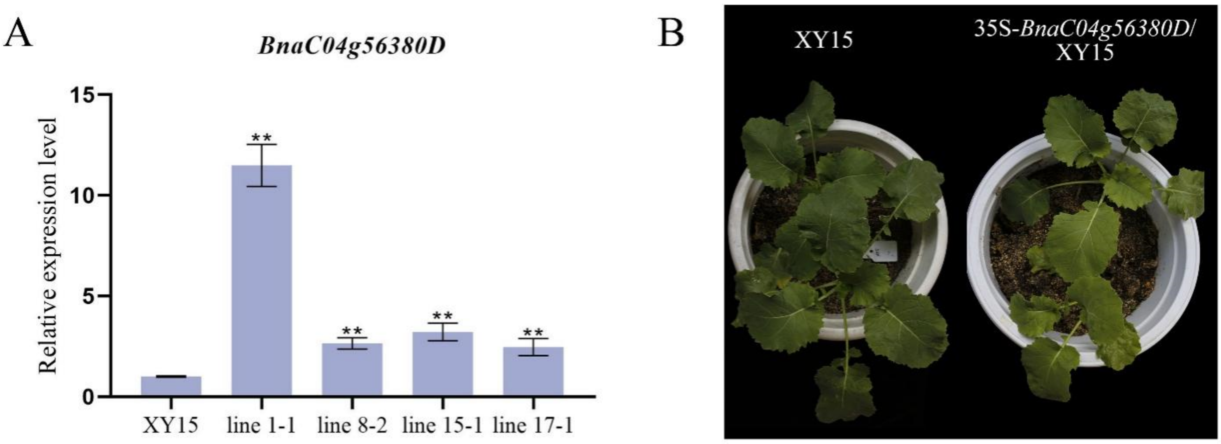

Supplementary Figure S4. *Bna*C04g56380D overexpression in XY15. (A) Relative expression levels of *Bna*C04g56380D in 35S-*Bna*C04g56380D/XY15. Error bars represent SD (** $p < 0.01$). (B) Phenotype of 35S-*Bna*C04g56380D/XY15 and XY15, all plants were grown on soil at 22°C in parallel and photographed when they were 120-days old.

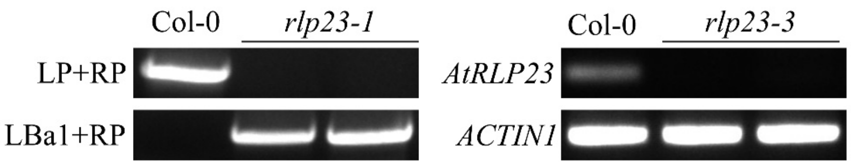

Supplementary Figure S5. Genotype identification of *rlp23-1* and *rlp23-3*. Genotype identification of *rlp23-1* is performed using three primers (LBa1+LP+RP) for SALK lines, where LP+RP for wild type should yield a product, LBa1+RP for homozygous lines will yield a band, and for heterozygous lines will yield both bands. Genotype identification of *rlp23-3* by Semi-quantitative PCR of *AtRLP23*, *ACTIN1* as the reference gene.

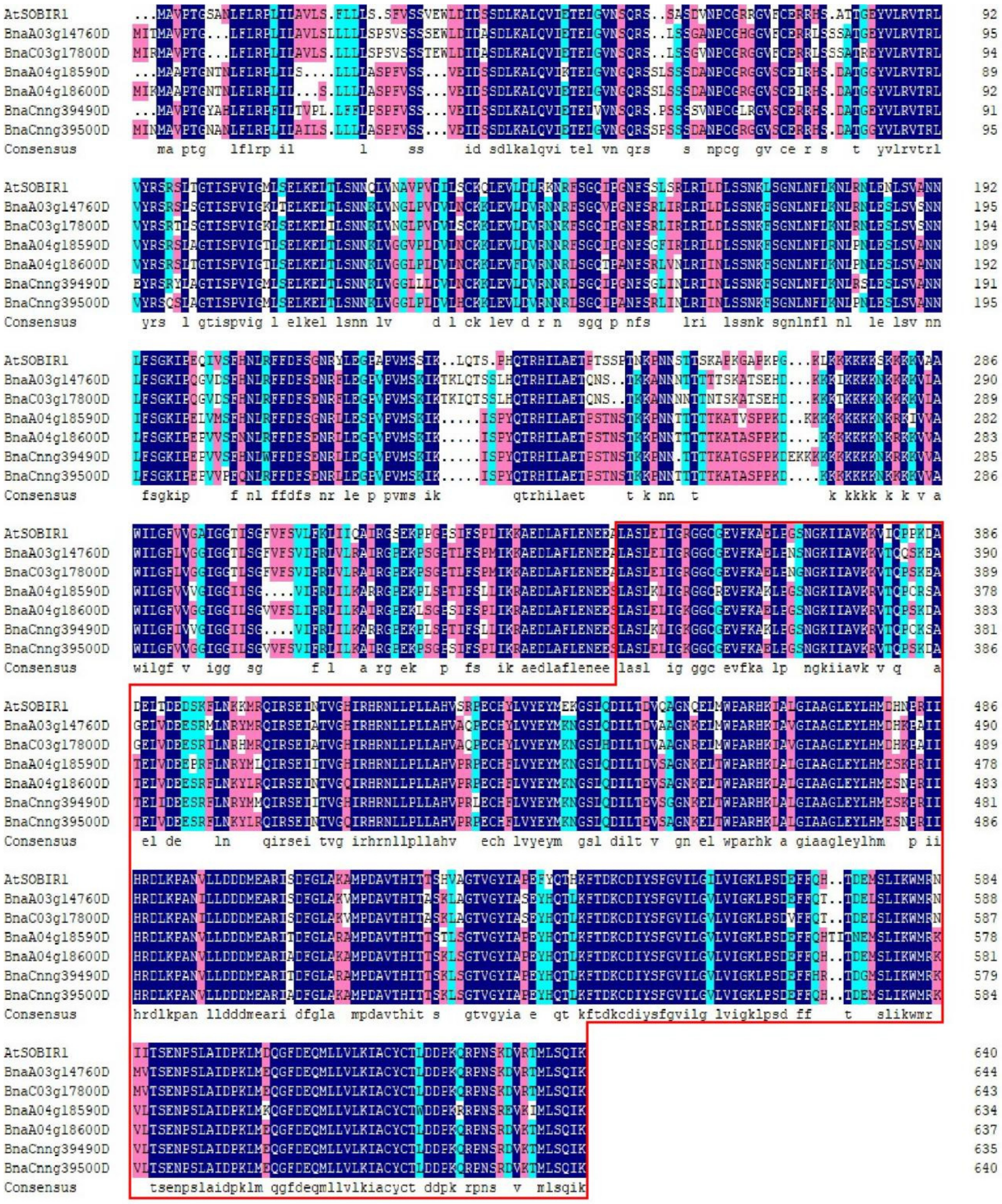

Supplementary Figure S6. Sequence alignment between *AtSOBIR1* and *BnaSOBIR1*. Amino acid sequences were derived from *At*SOBIR1, *Bna*A03g14760D, *Bna*C03g17800D, *Bna*Cnng39490D, *Bna*A04g18590D, *Bna*A04g18600D and *Bna*Cnng39500D. Red box: kinase domain, analyzed by PredictProtein.

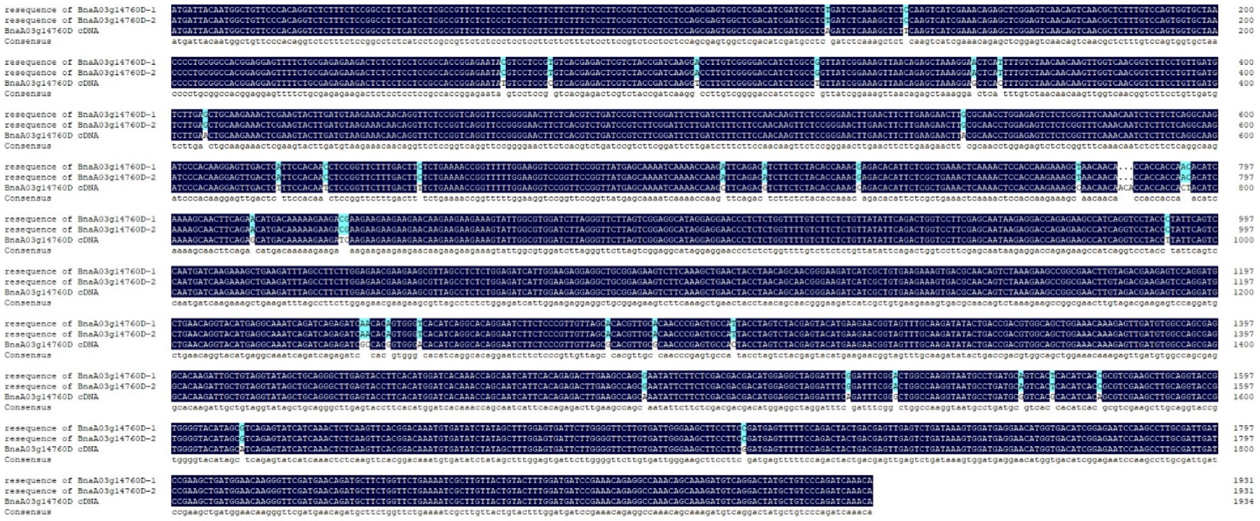

Supplementary Figure S7. Alignments of the resequencing sequence of *BnaA03g14760D.* The first and second rows were re-sequencing results of *BnaA03g14760D* (the amplified sample was derived from RNA of XY15), the third row was cDNA sequences of *BnaA03g14760D* in the database (*BnaA03g14760D* contains only one exon, and the cDNA and DNA sequences are identical).

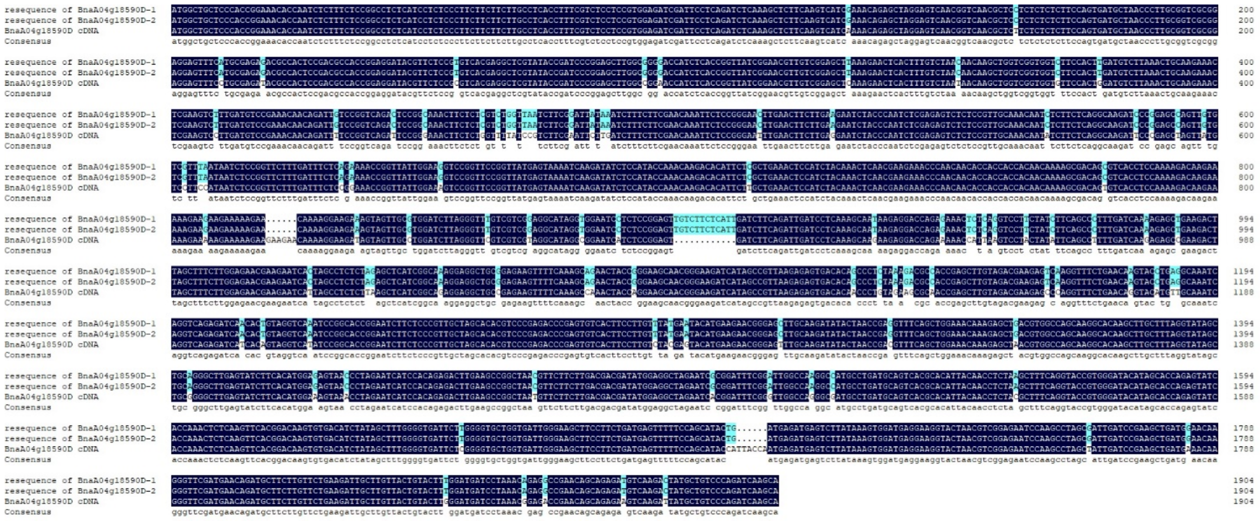

Supplementary Figure S8. Alignments of the resequencing sequence of *BnaA04g18590D.* The first and second rows were re-sequencing results of *BnaA04g18590D* (the amplified sample was derived from RNA of XY15), the third row was cDNA sequences of *BnaA04g18590D* in the database (*BnaA04g18590D* contains only one exon, and the cDNA and DNA sequences are identical).

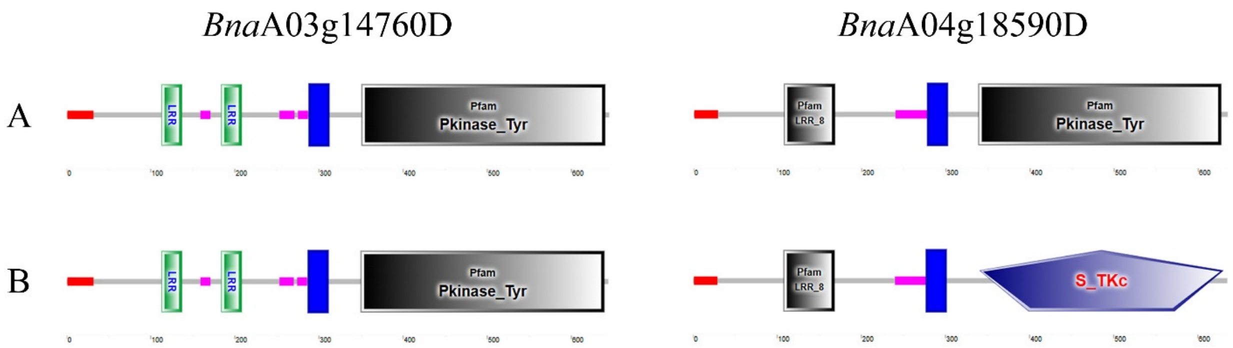

Supplementary Figure S9. Secondary structure of *Bna*A03g14760D and *Bna*C03g17800D. (A) Prediction results for the *Bna*A03g14760D and *Bna*C03g17800D sequence obtained from the Ensembl plants. (B) Prediction results for the *Bna*A03g14760D and *Bna*C03g17800D sequence cloned from XY15 and resequenced. Red box: signal peptide, green box/grey square box: represent the leucine-rich repeats (LRR) domain, purple box: low complexity region, blue box: transmembrane domain, and grey rectangle box/blue pentagonal box: kinase domain.

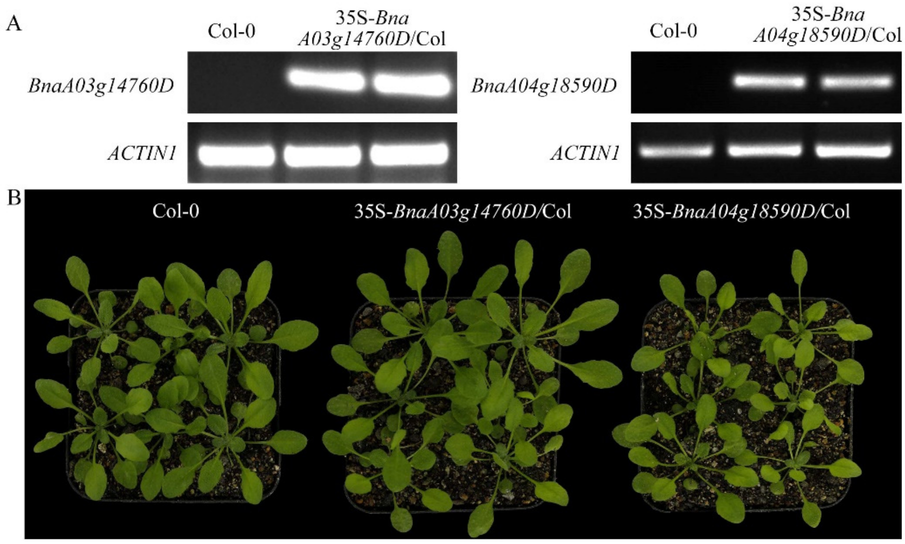

Supplementary Figure S10. *BnaSOBIR1* overexpression in Col-0. (A) Genotype identification of overexpression lines is performed using semi-quantitative PCR of *BnaSOBIR1*, *ACTIN1* as the reference gene. (B) Phenotype of 35S- *BnaSOBIR1*/Col and Col-0, All plants were grown on soil at 22°C in parallel and photographed when they were 4 weeks old.

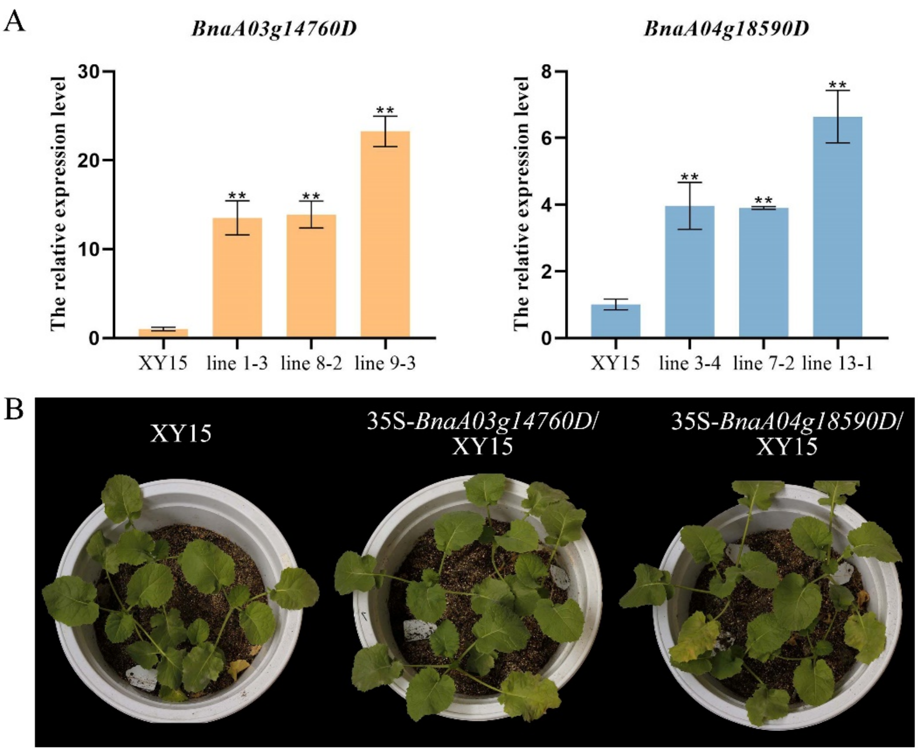

Supplementary Figure S11. *BnaSOBIR1* overexpression in XY15. (A) Relative expression levels of *BnaSOBIR1* in 35S-*BnaSOBIR1*/XY15. Error bars represent SD (** $p < 0.01$). (B) Phenotype of 35S-*BnaSOBIR1*/XY15 and XY15, all plants were grown on soil at 22°C in parallel and photographed when they were 120-days old.

2 Supplementary Table

Supplementary Table S1. Primers for research.

Primer name	Primer sequence	Primer function
RLP23-qF	ttcactggtcccattcctcc	qRT-PCR
RLP23-qR	tgtccgtaaagaggcaccat	
56380-qF	tggcaagctcgaaggtgaaa	
56380-qR	aagcaacactccaggtccata	
SOBIR1-1-qF	tcacatggatcacaaaccagc	
SOBIR1-1-qR	gccagcccgaaatctgaaat	
SOBIR1-2-qF	ggaagcaacgggaagatca	
SOBIR1-2-qR	gggtctcgggacgtgtgcta	
BnaBAK1-qF	catggctaatggaagtgttgc	
BnaBAK1-qF	atgaatgatctttgggtcgca	

Primer name	Primer sequence	Primer function
UBC9-qF	gcatctgcctcgacatcttga	
UBC9-qR	gacagcagcaccttggaaatg	
AtSOBIR1-qF	aaccaccaggtccttccata	
AtSOBIR1-qF	ttagttcatcggcgtctttag	
AtBAK1-qF	gcttcggcttcgtggattt	
AtBAK1-qR	cccaacgcaatacgctgtc	
YNE-RLP23-F	cacgggggactctagaggtaccatgtcaaaggcgcttttgc	BiFC
YNE-RLP23-R	ccatgtcgacactagtggatccacgctttctgcgtttat	
YNE-56380-F	cacgggggactctagaggtaccatgtctgaatcccgtgtgc	
YNE-56380-R	ccatgtcgacactagtggatccacggtttctgcgattg	
YCE-14760-F	ggagagaacactgcagggtaccatgattacaatggctgt	
YCE-14760-R	acatgtcgacactagtggatccgtgtttgatctgggaca	
YCE-18590-F	ggagagaacactgcagggtaccatggctgctcccaccggaaac	
YCE-18590-R	acatgtcgacactagtggatccgtgcttgatctgggacagcataat	
YCE-NEP2-F	ggagagaacactgcagggtaccatggttgcctttgcc	
YCE-NEP2-R	acatgtcgacactagtggatccgaaactactagcctt	
YCE-nlp24-F	gagagaacactgcagggtaccatgggactgatgtattcc	
YCE-nlp24-R	acatgtcgacactagtggatccttcccagtcgtggcg	
56380-STE-F	tctattttatgtaatggccattacggccatgtctgaatcccgtgtgc	Y2H
56380-STE-R	cgaattcctgcagatggccgaggcggccccacggtttctgcgattg	
pPR3N-14760-F	ggtatcaacgcagagtggccattacggccatgattacaatggctgt	
pPR3N-14760-R	tatcgaattctcgagaggccgaggcggccctagtgtttgatctgggaca	
pPR3N-18590-F	ggtatcaacgcagagtggccattacggccatggctgctcccaccggaaa	
pPR3N-18590-R	cgaattctcgagaggccgaggcggccctagtgcttgatctgggacagca	
